# On the Treatment of Albuminuria in Children

**Published:** 1864-10

**Authors:** 


ON THE

### TREATMENT OF ALBUMINURIA IN CHILDREN.

[At a recent meeting of the Royal Medical and Chirurgical Society a paper was read on this subject by Dr. W. H. Dickinson, of which we copy an abstract and the discussion following it, from the *Medical Times and Gazette.*]

The granular kidney appears to be unknown in childhood. The only form of disease which produces albuminuria at this period of life is that which produces enlargement of the kidney and gives it a smooth mottled exterior. This is, in fact, a renal catarrh. The tubes become obstructed by an excess of their own epithelial growth, and hence arise all the evils of the disease. If only there is a free escape for the contents of the tubes the vascularity of the gland will be relieved by secretion, and the disorder will soon be at an end. The principle of treatment must be to send as much water as possible through the organ. This fluid is devoid of irritating properties, and probably passes through the gland rather by filtration than true secretion. With these views the patients were restricted to a fluid diet. They took from two to four pints of distilled water daily, and small doses of the infusion of digitalis. When the active symptoms had subsided iron was given. Twenty-six cases were adduced in which this treatment had been pursued. Twenty-two recovered completely; three were lost sight of while improving, but while still having a small quantity of albumen in the urine; one case did badly, and eventually died under other treatment. Many of the cases were of great severity. These results appear better than those afforded by other methods. Among the in-patients at the Children's Hospital otherwise treated, 11 died out of 39; and of 69 cases treated by Dr. Miller in Dispensary practice, 8 died. It was found that on an average the little patients were restored to apparent health in 30 days, while 15 days more were needed to get rid of the last traces of albumen. The use of the water did not seem in any case to increase the dropsy, but the contrary. It was usual, however, when the swelling was great to let the digitalis set up a certain amount of diuresis before ordering the full quantity. The subsequent use of iron was believed to correct the effects of the disease, without influencing the disease itself. On the occurrence of secondary disorders, such as convulsions, or acute inflammatory attacks, it was argued that the treatment of the renal mischief should be sedulously persisted in, with such additions as might be called for. The anæmic state of brain in uræmic convulsions, and their frequent occurrence after the exhaustion of diarrhœa or vomiting, were urged as reasons for abstaining from depressing remedies. A case was cited in which, under these circumstances, small doses of opium had been used successfully. A case was also given in which acute pleurisy had passed off under the use of only local measures. The paper professed to deal only with the albuminuria of childhood.

Dr. Fuller had had opportunities of witnessing the author's treatment, but chiefly in adults, in which it was not so successful as in children. In adults the renal affection was of longer standing. He had tried it in a child in private with success. It was successful in the dropsy of adults, but not so uniformly so as in dropsy in children after scarlet fever. If Dr. Dickinson's views were correct it was easy to understand that it should be more useful in children than in adults.

Dr. Basham said there were many points of practical value in the paper, and yet one or two things for comment. First, in cases where the renal affection followed scarlet fever let the treatment be what it might, the majority of patients did well. No doubt the diuretic influence of water was beneficial, but he thought the theory brought forward was open to a difference of opinion. He ventured to think that there was not merely a blocking up of the tube, but also some change of the gland-cells—they had become effete cells, and accordingly were thrown off, and these, of course, obstructed the tube. But we know that nature tries to get rid of them, and no doubt diluents are of help in this way. He should, however, think the natural tendency of such cases to do well had more to do with recovery than the treatment.

Dr. Hillier had tried Dr. Dickinson's plan, and had found it scarcely so satisfactory. He (Dr. Hillier) agreed with Dr. Basham, that such patients, however treated, if seen early, would nearly always do well. His plan of treatment was to keep them in bed, to use hot-air baths, to give purgatives and good diet. Occasionally he had under care a severe case, and then had tried the water plan, but had been obliged to resort to other means. Perhaps, however, he had been too timid. In one case purpuric symptoms came on. There was considerable hæmaturia and epistaxis. With the water treatment the patient did worse, but when it was given up and gallic acid administered, the patient recovered.

The Author, in reply, said that he was quite aware that the majority would get well if left alone, but a great number would not. He had been able to collect a large number of cases of deaths in the post-mortem books of St. George's Hospital. In reference to Dr. Basham's remarks, he said the first changes were, he believed, in the quantity and not in the quality of the epithelium. In reference to Dr. Hillier's want of success, he would fall back on the explanation which the Dr. himself had suggested, viz., that the treatment was not persevered in. The only fatal case in the paper was the one in which the vapor bath had been used.

Mr. Bainbridge asked if the treatment would have been equally successful without the digitalis? He had seen many such cases during the last month, and the patients generally were thirsty, and, as a consequence, without any special direction, drank a great deal of water.

The Author said that the water treatment had been tried alone in three cases.

Dr. Stewart related a case of dropsy following scarlet fever occurring in a woman who had recently been confined. Suddenly convulsions came on, followed by unconsciousness. Cupping the loins, and other measures were followed by success, and he thought it a case in which the use of diluents would not have led to an equally favorable result.

Dr. Basham said that he thought that if any pathologist well versed in the use of the microscope would examine the gland-cells of the kidney of a child who had died by accident, and then those of one who had died from renal disorder, he would find a well-marked difference. In the latter the cell was cloudy, and the nucleus obscure. It had become an effete and imperfect cell.

Dr. Fenwick said that he had examined the mucous membrane of the stomach in scarlet fever patients, and had found changes in the epithelium there as well as in that of the kidney.

In reply, Dr. Dickinson said that no doubt there was a change in the cells, but not primarily.—*Boston Med. and Surg. Journal.*

